# Uterine Transplant Optimization From a Preclinical Donor Model With Controlled Cardiocirculatory Arrest

**DOI:** 10.1097/TXD.0000000000001735

**Published:** 2024-12-10

**Authors:** Emma Loiseau, Benoit Mesnard, Sarah Bruneau, Carla De Sousa, Stéphanie Bernardet, Jeremy Hervouet, David Minault, Stephan Levy, Antoine Le Gal, Ludivine Dion, Gilles Blancho, Vincent Lavoue, Julien Branchereau

**Affiliations:** 1 CRT2I UMR 1064, Nantes Université, CHU Nantes, INSERM, Centre for Research in Transplantation and Translational Immunology, Nantes, France.; 2 Department of Gynecology, Nantes University Hospital, Nantes, France.; 3 Department of Gynecology, Rennes University Hospital, Hôpital Sud, Rennes, France.; 4 Department of Radiology, Nantes University Hospital, Nantes, France.; 5 Irset - Inserm UMR_S 1085, Rennes, France.; 6 Department of Urology and Transplantation Surgery, Nantes University Hospital, Nantes, France.

## Abstract

**Background.:**

Uterus transplantation from deceased donors offers a promising solution to the organ shortage, but optimal preservation methods are crucial for successful outcomes. Our primary objective is to conduct an initial assessment of the contribution of oxygenated hypothermic perfusion in uterine transplantation.

**Methods.:**

We performed a preclinical study on a porcine model of controlled donation after circulatory death (60 min warm ischemia). Ten uterus grafts were preserved for 12 h using static cold storage or hypothermic machine perfusion (VitaSmart device, perfusion pressure at 15 mm Hg). Subsequently, they were reperfused using ex vivo normothermic machine perfusion (Liverassist, perfusion pressure at 30 mm Hg) with oxygenated autologous blood to assess early ischemia/reperfusion injury. Not only resistance index assessment and oxygenation evaluation but also immunochemistry and gene expression analysis were performed.

**Results.:**

This study demonstrates the feasibility of using hypothermic machine perfusion for uterine graft preservation, showing improvements in reperfusion capacity (decrease of resistance indexes; *P* < 0.0001) and tissue oxygenation (higher oxygen level) compared with static cold storage.

**Conclusions.:**

These findings provide valuable insights for further research and refinement of uterine transplantation procedures.

Uterine transplantation (UTx) has emerged as a novel surgical treatment, initially with live donors^1^ and later with deceased donors.^2^ Although most UTx procedures have used living donors (LDs),^3^ recent advancements have explored the potential of deceased donors. These encouraging results allow women with absolute uterine factor infertility, such as Mayer Rokitansky Küster Hauser syndrome and severe forms of unification defects, to access parenthood.^4^ This procedure was still considered an experimental fertility treatment, and many aspects of the procedure can be improved or refined. Although LDs offer advantages such as shorter cold ischemia periods, they also entail higher complication rates.^5-7^ UTx from deceased donors presents distinct advantages, including access to younger donors, lower costs, and equitable transplant access.^2,8-14^

On a national scale, in France, controlled donations after circulatory death (cDCD), corresponding to the context of a decision to limit or discontinue treatment, are very numerous, accounting for 11% of donations from 44 procurement centers. In addition, the program for organ cDCD offers excellent results in kidney and liver transplantation.^15,16^ In this case, the prognosis for success in graft seemed to depend on ischemia/reperfusion injury.^17^ The present study addresses the potential of using uterus grafts from cDCD to alleviate organ shortage.

The uterus has shown resilience to warm and cold ischemia, but optimal preservation methods remain unclear. Previous studies have investigated various durations of warm, ranging from 1 h to strictly <5 h (in human’s uteri)^18^ to strictly <5 h (in rat and ewe’s uteri),^19,20^ and cold ischemia tolerated by uterine transplants, ranging from 12 h (in human, macaque, rat and ewe’s uteri)^18-21^ to 24 h (in human, sheep, and mouse’s uteri).^11,22-25^ However, static cold storage (SCS) has been the primary preservation method described.

Ex vivo normothermic machine perfusion has emerged as a valuable tool for assessing uterine transplant reperfusion, offering advantages such as easy access to biological samples and reproducibility.^15,22,26-28^

This study introduces an innovative technique for preserving uterine transplants using hypothermic machine perfusion (HMP) in a porcine cDCD model.

The objective of this hypothermic perfusion is to optimize preservation conditions and mitigate ischemia/reperfusion injury. Following preservation, uterine grafts undergo ex vivo normothermic reperfusion to evaluate reperfusion capacity and assess parameters such as oxygen availability, oxidative stress, inflammation, and tissue damage.

## MATERIALS AND METHODS

### Uterus Harvest Procedure

The study used large female White-breed pigs weighing approximately 150 kg. On purpose, sows that already had litters were chosen to meet the criteria for validated acceptability of transplants in humans. The surgical procedure was based on the previously described uterine retrieval technique during multiorgan retrieval,^12^ adapted to porcine anatomical variations and simplified because of the absence of planned retransplantation in the study.

**FIGURE 1.**
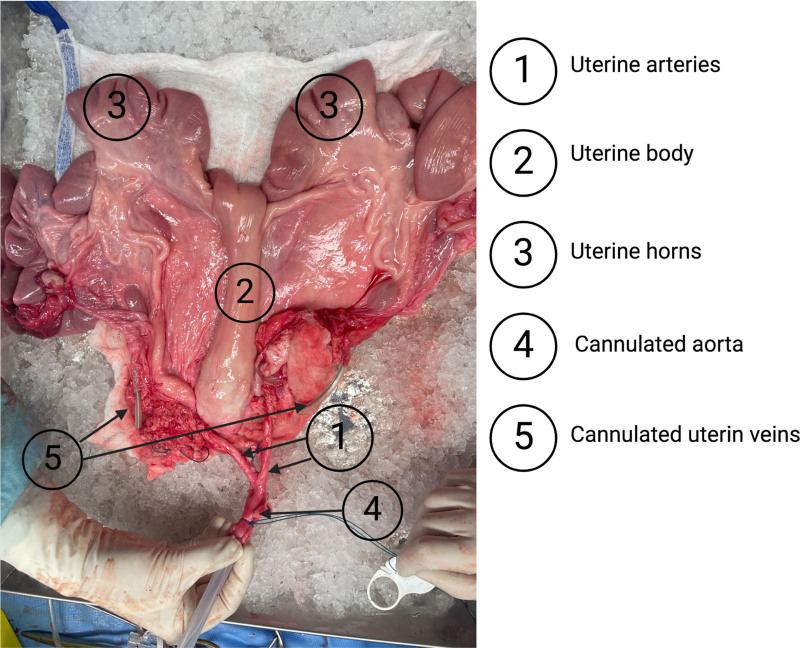
Explant uterus prepared for hypothermic perfusion.

Due to ethical reasons and to perform an optimal management of controlled cardiocirculatory arrest, the surgical procedure was initiated under general anesthesia. The first step was to perform an exenteration of the digestive tract to properly approach the retroperitoneal space. The subsequent procedural phase involved the implementation of thoracic cannulation through the left thoracic cavity, facilitating the thorough irrigation of abdominal organs for optimal efficacy. Unfractionated heparin 300 mUI/kg was injected before the reproduction of cardiocirculatory arrest, marking the onset of warm ischemia, by intravenous injection of pentobarbital and wedging of the aorta at the thoracic level, enabling the animal to be euthanized. Meanwhile, blood was drawn from the cannulated vena cava for subsequent reperfusion of the organ. At the end of 60 min of warm ischemia, the abdominal organs were flushed with IGL-1 solution at 4 °C via the aortic cannula, where the inferior vena cava was unloaded and the abdominal cavity iced. Next, dissection of the disc aorta began 7 cm upstream of the bifurcation of the uterine arteries, with ligation of all arteries originating from the posterior aspect of the aorta. After careful identification of the feeder vessels, the external iliac arteries were tied. We proceeded with vesicouterine detachment, followed by retrograded dissection of the ureters, dissection of the rectovaginal wall, and passage off the parametrium. At last, the explanted uterus was prepared on the back table with cannulation of the distal aorta and large-caliber uterine veins in the parametrium. The organ was flushed with IGL-1 from the aortic cannula to inspect for arterial leaks and complete uterine flushing (Figure 1).

### Preclinical Experimental Groups

Uterine transplants were preserved for 12 h using either SCS or hypothermic machine perfusion (HMP). We chose a 12-h cold ischemia period because the uterus demonstrated good resilience to cold ischemia in preliminary studies, and we wanted to be as discriminating as possible between the 2 preservation methods.^11,18-25^ Each group was composed of 5 uteri. We decided to rely on solutions already approved for perfusion in other organs, tailored for use on perfusion machines, and approved for renal transplantation. For transplant preservation in the SCS group, we manipulated the Vitalpak EVO.

For the preservation of transplants in the HMP group, VitaSmart machine perfusion system was chosen. The simple perfusion system of the VITAsmart machine, intended for liver perfusion, allowed us to configure and enable real-time perfusion monitoring and temperature parameters and provided for external oxygen enrichment of the circuit. In addition, this machine was compatible with the size of the organ studied and provided external oxygen enrichment of the circuit, making it suitable for a feasibility study. The uterine transplant was perfused anterogradely by the machine via a cannula placed on the distal aorta. The flow rate adjusts according to the chosen pulsatile pressure target. No study has evaluated UTx on a HMP. We defined a perfusion pressure close to that of the pancreas (15 mm Hg), given the same size of their feeder vessels in our animal model. The optimal temperature defined was 4 °C, achieved by external cooling of the liquid surrounding the organ and monitored by an immersed thermal sensor. The temperature was based on the thermal targets used in organ preservation. The perfusion solution was enriched with oxygen at 4 L/min.

**FIGURE 2.**
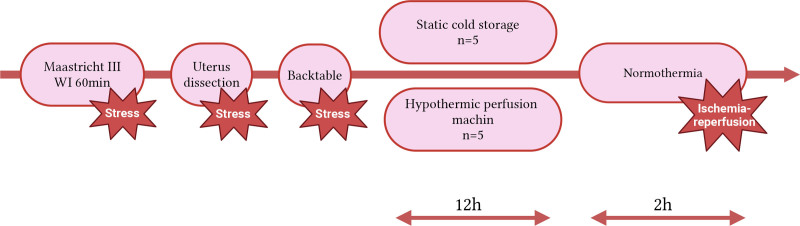
Flow diagram of the paths of procurement, storage, and reperfusion. WI, warm ischemia.

Following hypothermic preservation, uterine grafts underwent ex vivo normothermic reperfusion using autologous blood. We used a Liverassist machine. This machine is validated for liver transplantation and applicable to UTx (vessel caliber and machine capacity). It features perfusion pathways for the portal and hepatic networks. We had to adapt the device from a double to a single circuit, using the hepatic pathway with pulsatile flow. The machine was set for a target blood warmth of 37 °C and an optimal perfusion pressure of 30–35 mm Hg, until achieving satisfactory venous return. The instituted oxygen supply was 4 L/min to achieve an average blood oxygenation of 60 mm Hg. This machine assessed the organ’s reperfusion capacity, as measured by resistance indexes. The organ was reperfused with autologous blood collected during the warm ischemia phase, enriched with heparin (5000 IU units of heparin sodium), vasodilator (1 g of Nicardipine), and antibiotics (1 g of amoxicillin and clavulanic acid). Three liters were needed to compensate for the dead volumes of the ex vivo normothermic machine circuit and ensure satisfactory organ perfusion (Figure 2).

### Measurement of Tissue Oxygen Pressure

Po_2_ was measured continuously using the OxyLite Pro XL system (Oxford Optronix Ltd, Abingdon, United Kingdom). The technology is suitable for measuring oxygen in the physiological range and is sensitive to hypoxic conditions. Intratissue probes were placed in the uterine body (deep: 1 cm) and horn to assess oxygen availability in the muscular tissue throughout the experiment. The endothelium could not be evaluated during the experiment due to its anatomical location.

### Histology, Immunohistochemistry, and Gene Expression Analysis

**FIGURE 3.**
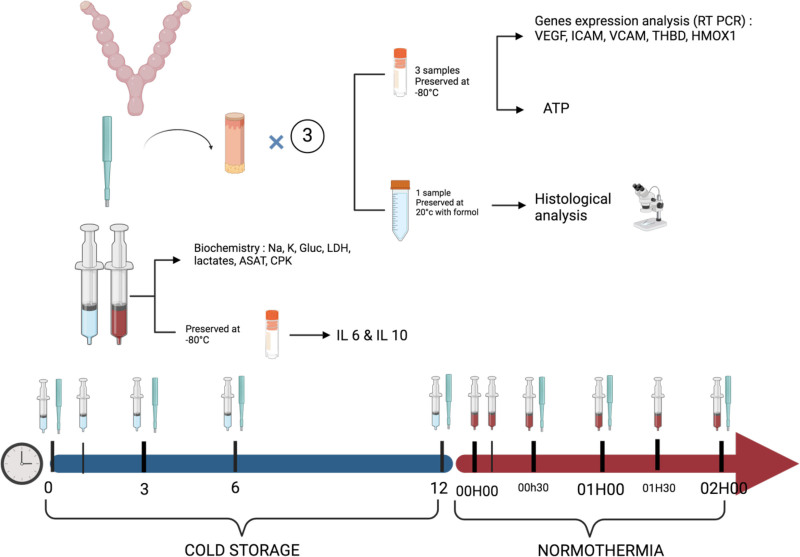
Points of data collection. ASAT, aspartate aminotransferase; CPK, creatinine phosphokinase; IL, interleukin; LDH, lactate dehydrogenase; RT-PCR, real-time polymerase chain reaction.

Data collection points are resumed in Figure 3.

Tissue biopsies were obtained during hypothermic and normothermic phases for histological and molecular analysis, using a 3-mm diameter dermal biopsy punch. Biopsies were taken at the antimesial edge of the vessels on the horn and on the anterior or posterior surface of the uterine body. Some biopsies were preserved in a 10% formalin solution; others were frozen directly at –80 °C, whereas the remainder were embedded in Tissue-Tek cassettes containing a water-soluble solution (Tissue-Tek OCT cryo embedding compound). This cassette was immersed in an isopenthane solution cooled by dry ice.

At this step, formalin‐fixed tissue samples were embedded in paraffin, sectioned at 5 μm and then stained for hematoxylin and eosin and 4′,6‐diamidino‐2‐phenylindole (25 μg/mL) according to standard procedures.

ATP has then been measured by luminescence detection using the luciferase technique with the ATP Bioluminescence Assay Kit HS II (Cat#11699709001; Sigma Aldrich, Saint-Louis, MO).

Succinate assay was performed using a colorimetric technique with the Succinate Colorimetric Assay Kit (Cat#MAK184; Sigma Aldrich).

Analysis of endothelial gene expressivity by real-time polymerase chain reaction evaluated 5 endothelial genes: *ICAM-1 and VCAM-1* (main roles in immune cell adhesion and inflammation through endothelial activation), *VEGF* (role in angiogenesis), *HMOX-1* (protective role against oxidative stress), and *THBD* (inhibits thrombosis). mRNAs were extracted using the RNeasy MiniKitTM kit (Qiagen, reference No. 74106, Hilden, Germany), retrotranscribed using the Invitrogen kit, Fisher Scientific, and then analyzed for expression using specific probes (TaqMan Gene Expression Assays, Applied Biosystems) and TaqMan Universal Master Mix II, No AmpErase UNG (Applied Biosystems). The evolution of endothelial cell activation or inactivation is expressed in “fold change.” This metric represents the ratio between the expression level of a gene in a given organ preservation condition compared with the expression of the gene studied at the beginning of the hypothermic phase (T0) per biopsied area.

### Biochemical Analysis of Perfusate

During the hypothermic phase, fluid samples were withdrawn in the preservative solution in which the uterus was immersed. Part of the preservative liquid was stored at –80 °C until analysis, and the other part was centrifuged to promote preservation and then provided to the biochemical analysis laboratory at the end of the procedure.

Blood samples were taken directly from the cannulated uterine veins during the normothermic phase. Part of the liquid was stored at –80 °C, another portion was centrifuged in the same way as in the hypothermic phase, and the last segment was sent directly for gasometry.

Measurements of lactate, glucose, potassium, creatinine phosphokinase, hemoglobinemia, pH, Po_2_, Pco_2_, and lactate dehydrogenase during the experiment were performed on automated equipment in the biology laboratories of Nantes University Hospital.

The determination of proinflammatory (interleukin-6) and anti-inflammatory (interleukin-10) interleukins was performed by ELISA using the DuoSet ELISA kit (R&D Systems).

### Statistical Analysis

Statistical analyses were performed using GraphPad Prism software (version 9.1.1). Intratissue oxygenation levels during hypothermic preservation and normothermia, perfusion parameters, and biochemical results were compared using the ANOVA test and the multiple comparisons test after determining the air under the curve. Differences were considered statically significant when *P* < 0.05. To compare the areas under the curve, we need to assume a normal distribution of values.

### Ethics

The study was conducted in accordance with ethical guidelines and received approval from relevant authorities. The project referenced under number APAFIS #38037-2022071815101902 v3, submitted by the user establishment: Laboratoire Grands Animaux ITERT UMR 1064, approval number F44010, was authorized on October 31, 2022.

## RESULTS

### Index of Resistance to Uterine Transplant Reperfusion

**FIGURE 4.**
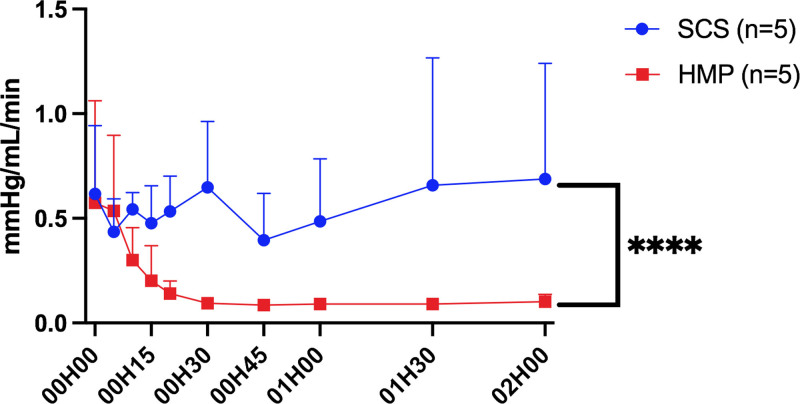
Evolution over time (hour) of machine resistance indexes during normothermic reperfusion according to the 2 study groups. In the SCS group, resistance indexes remained stable or even increased over time, whereas in the HMP group, they steadily decreased, becoming stable after 30 min of reperfusion. Each point corresponds to the mean ± SEM. HMP, hypothermic machine perfusion; SCS, static cold storage.

Resistance indices during ex vivo normothermic machine perfusion were significantly lower in the HMP group than in the SCS group (*P* < 0.0001; Figure 4).

### Oxygen Partial Pressure on Blood Gases

**FIGURE 5.**
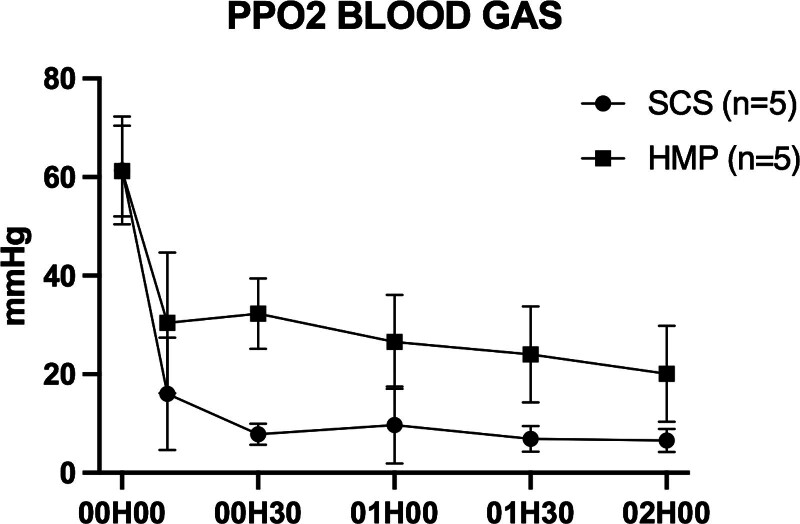
Evolution of Po_2_ on venous return during reperfusion over time (hour). Evaluation of Po_2_ on blood gas is based on samples taken from venous return cannulae. For each of the different biochemical marker measurements, the first measurement noted on the graphs “00H00” corresponded to prepared whole blood circulating at normothermia before organ placement. Each point corresponds to the mean ± SEM. HMP, hypothermic machine perfusion; SCS, static cold storage.

Po_2_ during ex vivo normothermic machine perfusion did not show significant differences between groups (Figure 5).

### Measurement of Tissue Oxygen Pressure

Intratissue Po_2_ probes were placed in the uterine body and horn in 6 and 7 cases, respectively, under general anesthesia with tracheostomy ventilation under FiO_2_ 50%. Under these conditions, the median oxygen partial pressure was 76 mm Hg for the body and 95 mm Hg for the horn. During the warm ischemia phase, Po_2_ decreased continuously over the first 20 min on the uterine body and from 30 min onward on the horn, reaching a minimum threshold value of 4 and 0.8 mm Hg, respectively.

**FIGURE 6.**
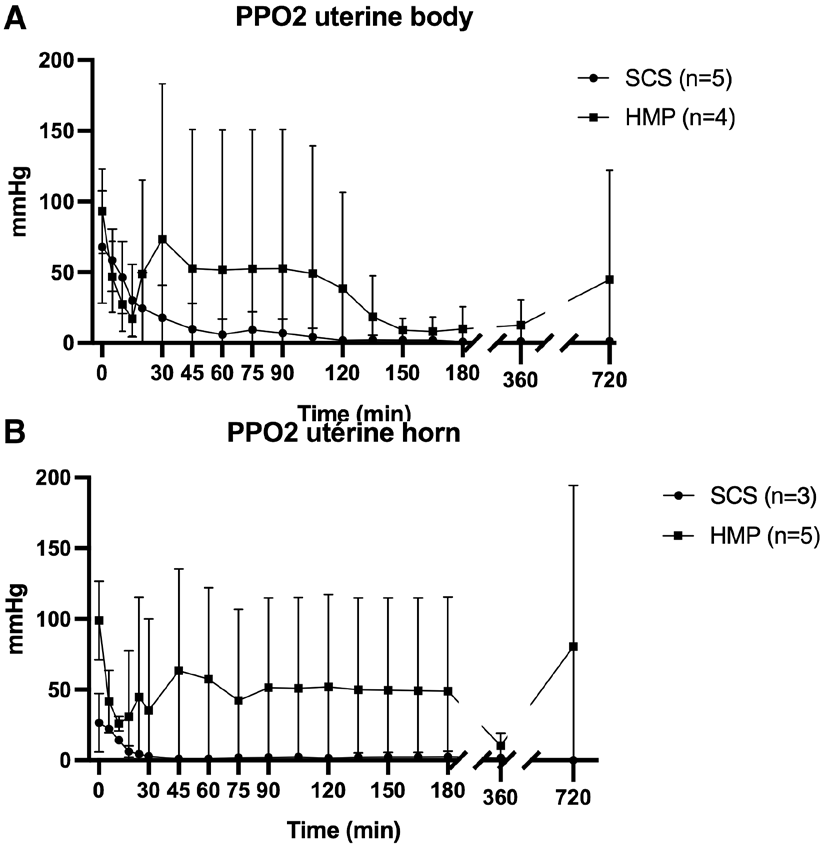
Evolution of tissue oxygenation during hypothermic preservation at body (A) and horn (B) levels over time (minute). In the SCS group, Po_2_ became zero within 1 h of hypothermic preservation. In the HMP group, the preservation medium circulating in the vessels was enriched with oxygen at 4 L/min for all organs. In the uterine horn and body, Po_2_ stabilized around 60 min after the start of hypothermic perfusion, tended to fall at 6 h and then rose again at 12 h. Each point corresponds to the mean ± SEM. HMP, hypothermic machine perfusion; SCS, static cold storage.

**FIGURE 7.**
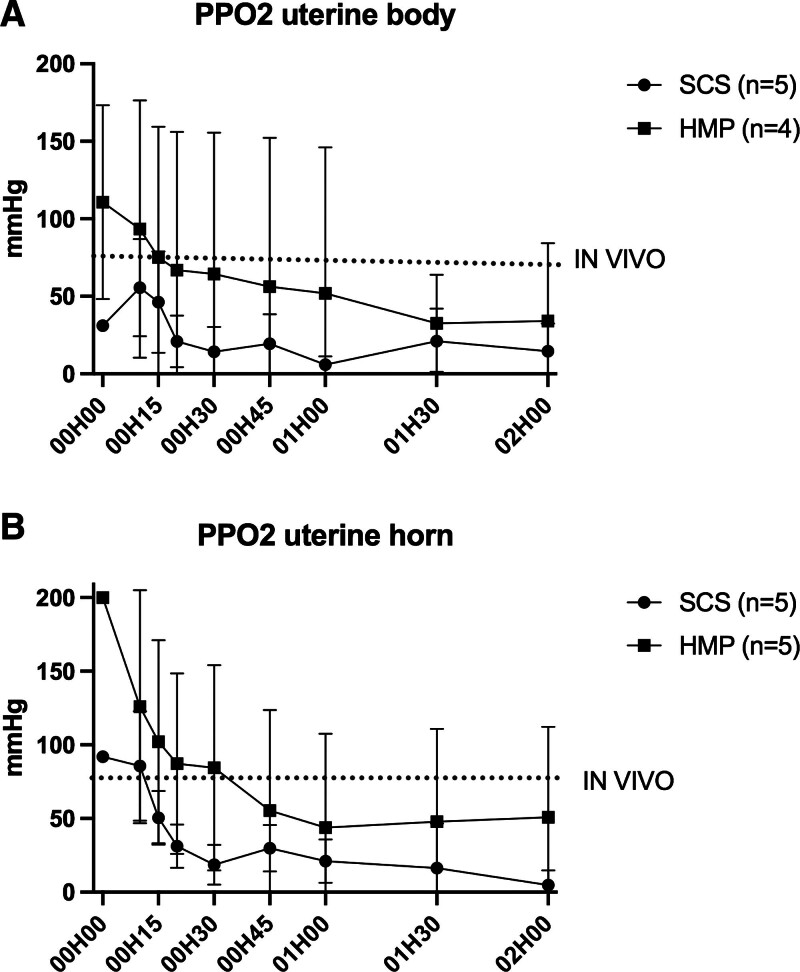
Evolution of tissue oxygenation during normothermia reperfusion at body (A) and horn (B) levels. The evolution of the curves shows a parallel decrease between the 2 groups over time (hour). Po_2_ values of the HMP group most closely resemble those measured in vivo. Each point corresponds to the mean ± SEM. HMP, hypothermic machine perfusion; SCS, static cold storage.

Intratissue Po_2_ showed increased oxygenation during HMP preservation compared with SCS, with variability between animals (Figures 6 and 7).

### Biochemical Analysis of Perfusate

**FIGURE 8.**
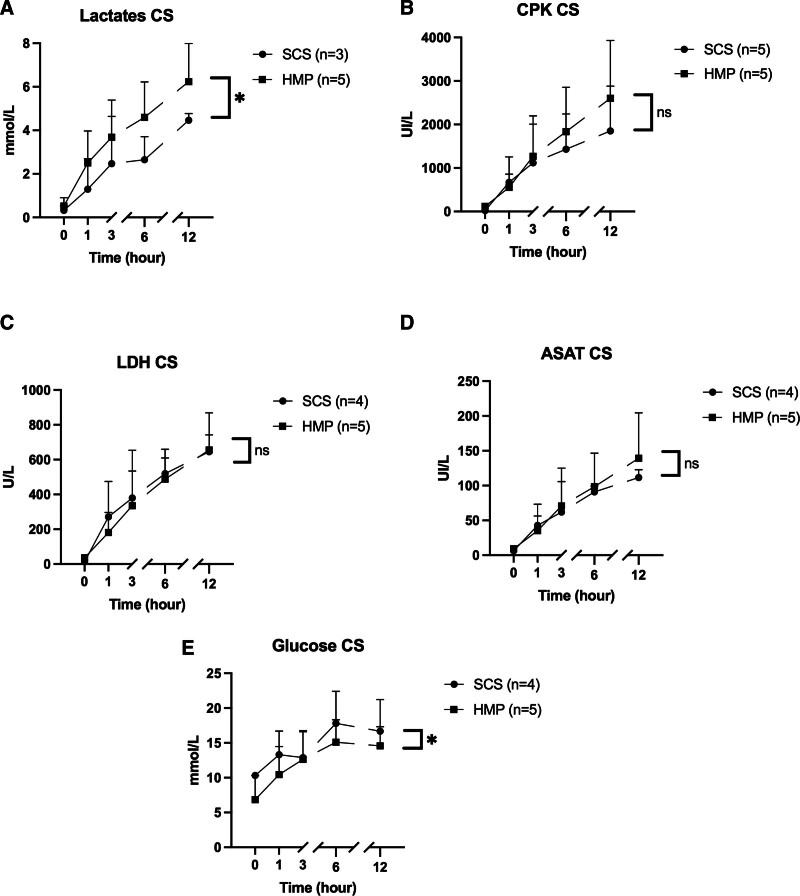
Evolution of perfusion fluid analysis during hypothermic preservation over time (hour): lactates (A), CPK (B), LDH (C), ASAT (D), and glucose (E). Each point corresponds to the mean ± SEM. ASAT, aspartate aminotransferase; CPK, creatinine phosphokinase; HMP, hypothermic machine perfusion; LDH, lactate dehydrogenase; SCS, static cold storage.

**FIGURE 9.**
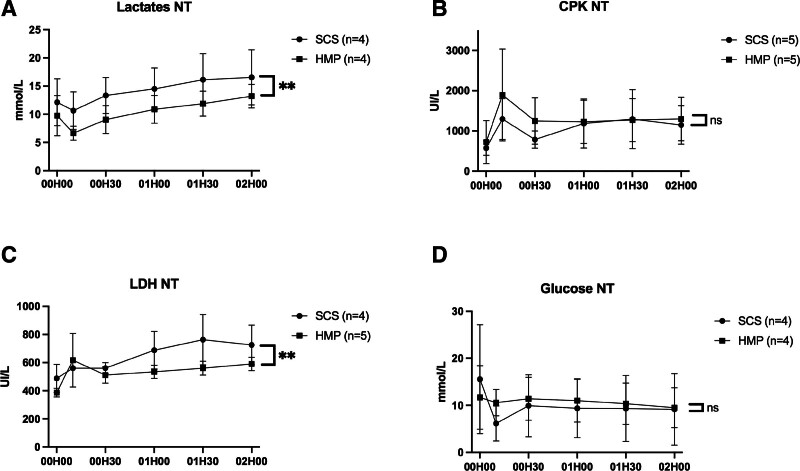
Evolution of perfusion fluid analysis during NT over time: lactates (A), CPK (B), LDH (C), and glucose (D). For each of the different biochemical marker measurements, the first measurement noted on the graphs “00H00” corresponded to prepared whole blood circulating at NT before organ placement. Each point corresponds to the mean ± SEM. Lactate levels increased in both groups, and remained parallel over time. LDH levels increased in both groups, with a greater rise in the SCS group. For the other markers assessed, there was no significant difference between the 2 groups. CPK, creatinine phosphokinase; HMP, hypothermic machine perfusion; LDH, lactate dehydrogenase; NT, normothermia; SCS, static cold storage.

Glucose and lactate levels were higher during hypothermic preservation in the HMP group (*P* = 0.0450 and 0.0130, respectively; Figure 8), whereas lactate and lactate dehydrogenase levels were reduced during normothermic reperfusion (*P* = 0.0019 and *P* = 0.0040, respectively; Figure 9). Some assays were not processed on a few participants due to excessively long sample collection and analysis times. The difference in glucose concentration measured at time T0 between the 2 groups can be explained by the sensitivity of glucose measurement to the conservation time before biochemical analysis.

### Assessment of Oxidative Stress

**FIGURE 10.**
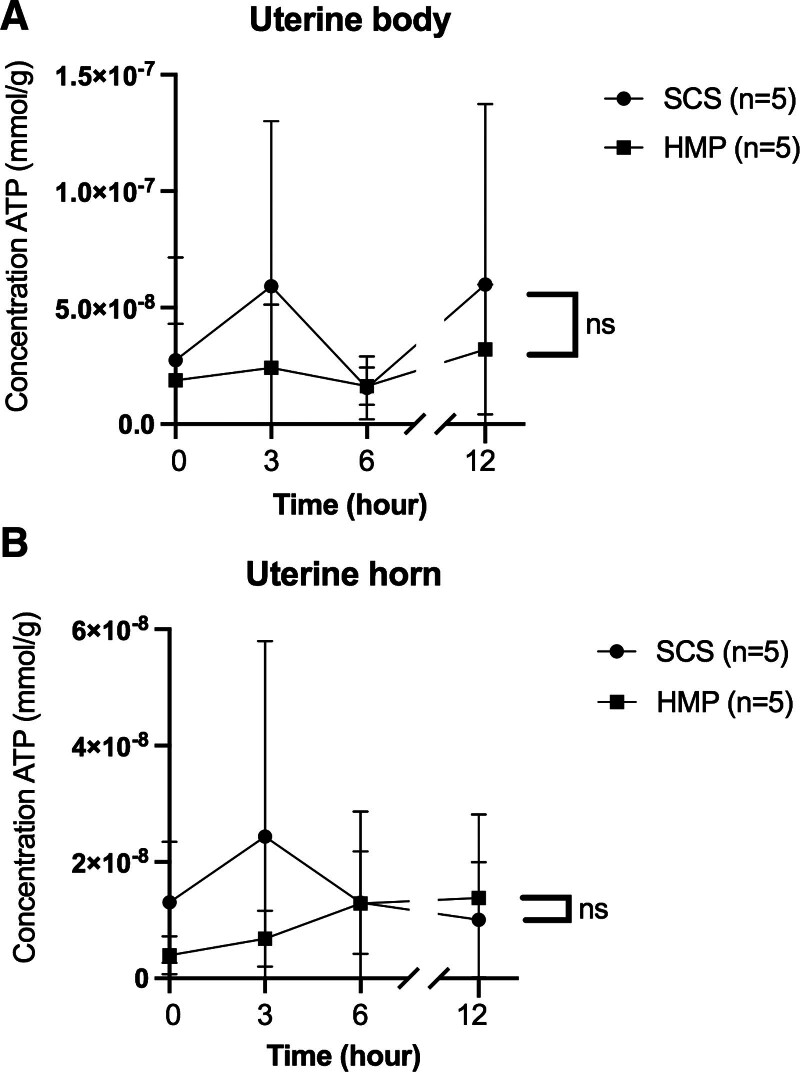
Evolution tissue ATP concentrations in the body (A) and uterine horn (B) during the hypothermic preservation phase over time (hour). Each point corresponds to the mean ± SEM. HMP, hypothermic machine perfusion; SCS, static cold storage.

**FIGURE 11.**
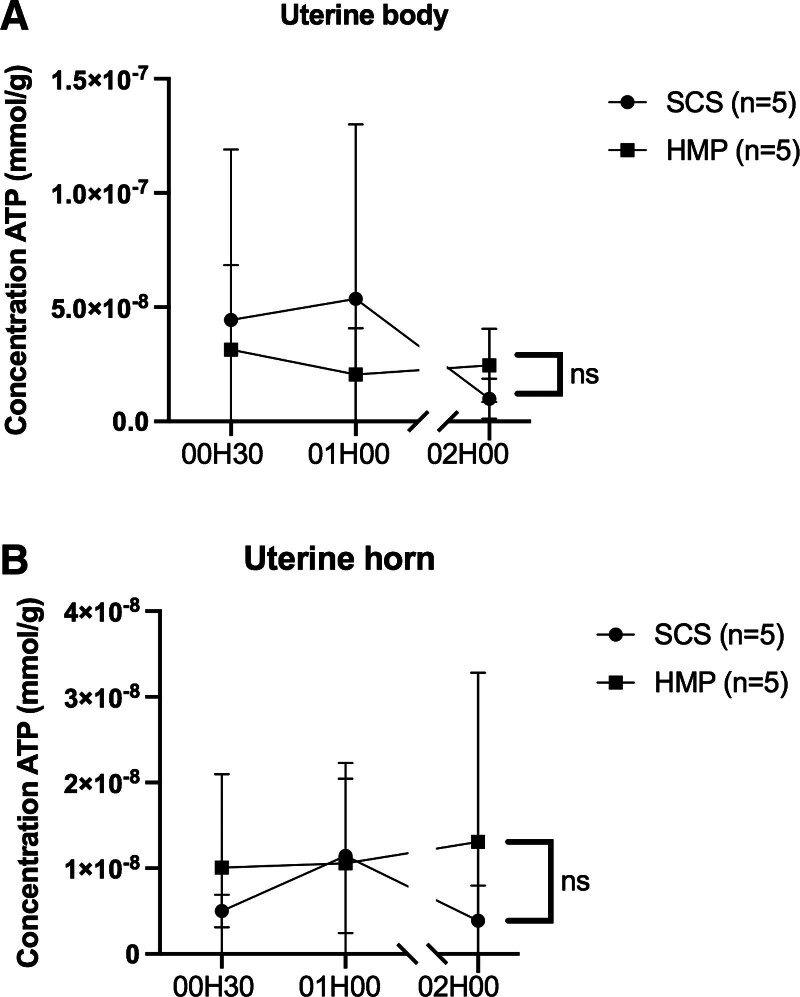
Evolution of tissue ATP concentrations of the body (A) and uterine horn (B) during normothermia over time (hour). Each point corresponds to the mean ± SEM. HMP, hypothermic machine perfusion; SCS, static cold storage.

**FIGURE 12.**
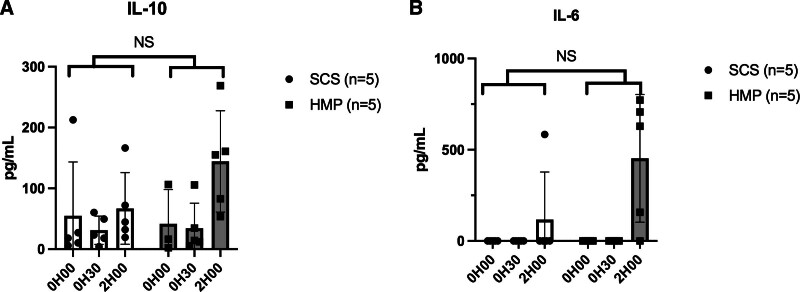
Evolution of concentration of interleukin 6 (A) and 10 (B) in the SCS and HMP groups during the reperfusion phase over time (hour). For each of the different biochemical marker measurements, the first measurement noted on the graphs “00H00” corresponded to prepared whole blood circulating at normothermia before organ placement. HMP, hypothermic machine perfusion; IL, interleukin; SCS, static cold storage.

Tissue ATP concentration and interleukin levels showed no significant differences between groups (Figures 10–12).

For the 5 genes studied (*VCAM, ICAM-1, VEGF, THBD, and HMOX-1*), the variability of gene expression does not seem to show any interpretable evolution between the 2 groups due to the low numbers (**Figures S1–S4, SDC,** http://links.lww.com/TXD/A721).

### Histology

**FIGURE 13.**
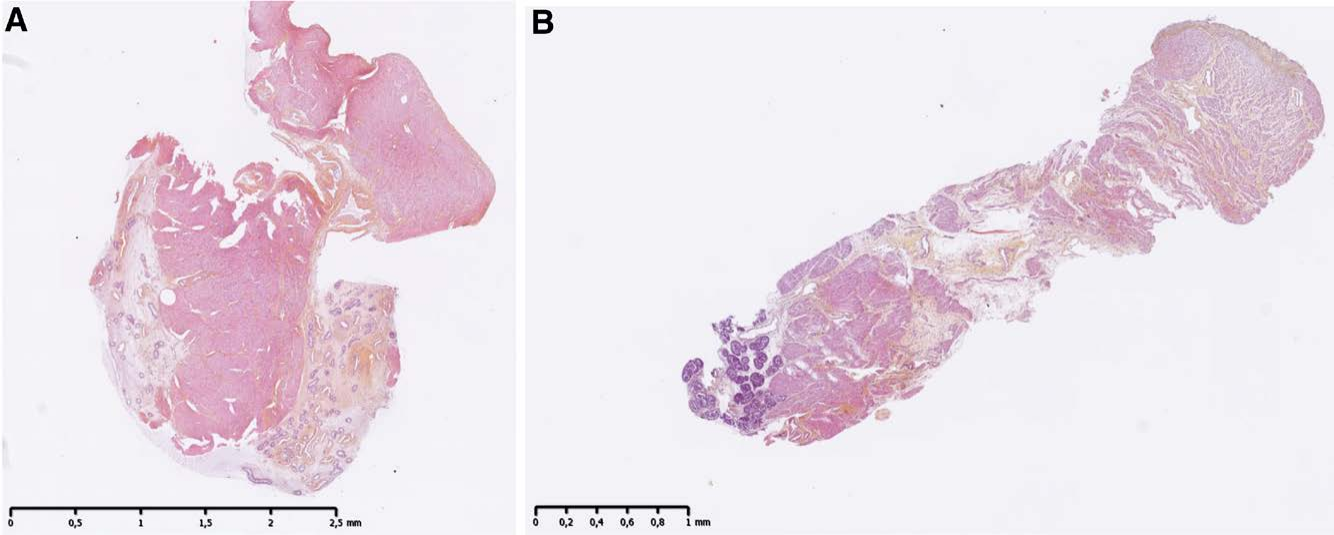
Histological sections of the uterine horn in the SCS group (A) and HMP group (B). SCS, static cold storage.

**FIGURE 14.**
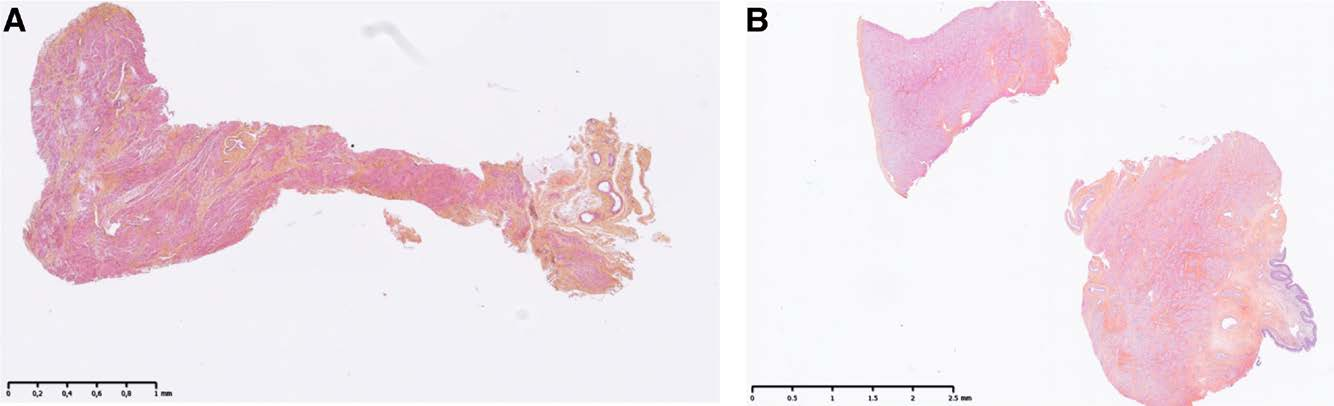
Histological sections of the uterine body in the SCS group (A) and HMP group (B). HMP, hypothermic machine perfusion; SCS, static cold storage.

Preliminary histological analysis showed increased interstitial edema in the HMP group compared with the SCS group after 12 h of preservation (Figures 13 and 14).

## DISCUSSION

UTx is still considered an experimental procedure and methodology refinement, and numerous critical details remain to be established. According to a review of literature published at the end of 2023, it is estimated that at least 71 UTx have been performed worldwide, two-thirds of them from LD. Among 62 UTx (LD or donation after brain death [DBD]), we can observe a surgical success rate (regular menstrual cycle) of 77%. According to the ISUTx registry, 19 live births from 16 recipients were registered (14/35 after LD UTx, 2/10 after DBD UTx). Three women after LD UTx gave birth to singletons twice.^4,29^ Given the high demand for uterine grafts, the shortage of grafts, and the lack of sufficient data for a reliable comparison of results between uterine grafts from living and deceased donors, it is now possible to use both types of donations, pending further results.^3^ According to the French Biomedicine Agency, the average age of DBD donors is 57.6 y, and 53.2 y for cDCD donors in 2022. Thus, cDCD-derived UTx would give patients greater access to grafts from younger donors. The challenge lies in the very short postmenopausal time frame for graft eligibility.

Some preclinical studies show certain surgical advantages of uterine retrieval from deceased donors. For example, a Japanese study published in 2018 evaluated different uterine retrieval modalities in primates using a live donor model (via autotransplantation) and a DBD model (via allotransplantation). The team describes a 2- to 4-fold faster vascular anastomosis time and a 2-fold faster average procedure time in the second model thanks to longer and wider feeder vessel access, making the retrieval mode more reproducible.^9^ However, other preclinical studies have highlighted the difficulties inherent in retrieved organs from deceased donors. A French study published in 2014 experimented with uterine retrieval as part of a multiorgan harvest from 14 DBD. Uterine retrieval was always performed in the last position, with a “warm” ischemia time (start of flush until explanation of the donor organ) of between 60 and 91 min, including a uterine retrieval time of 10–23 min.^11^ The uterus was removed after the rest of the abdominal organs to avoid the risk of bacterial contamination of other organs when the vagina is opened.^12,13^ Similarly, for the first successful UTx from a DBD in Brazil,^2^ the graft presented a “warm” ischemia of 50 min^8^ and a cold ischemia of 7 h 50^14^ compared with an average of 1–2 h for grafts from LDs.^6^ This incompressible “warm” ischemia time is a source of visceral suffering. These studies suggest that the uterus is an eligible organ for multiorgan retrieval, although the parameters for uterine graft preservation remain to be defined.

In the meantime, the HMP evaluated on several organs, such as the kidney and liver, offers encouraging results on improving early allograft lesions and postgraft outcomes in deceased donors after controlled cardiac-circulatory arrest.^30-33^

To our knowledge, this is the first study to investigate different modalities of uterine graft preservation in a cDCD model.

Our research has several strengths, as it shows the feasibility of uterine graft oxygenation during the hypothermic preservation phase, using judging criteria directly correlated with graft reperfusion capacity and other parameters assessing the quality of graft preservation. Thus, during the ex vivo normothermic machine perfusion phase, grafts from the HMP group showed significantly reduced resistance indexes and increased flow rates. In addition, intratissue probe results appear to show persistent oxygenation of the uterine body and horn during preservation with a HMP and preservation fluid enriched to 4 L/min oxygen. With an overall view of the results from the HMP group, reduced resistance indices, and assessments of intratissue oxygen partial pressures appearing to increase, we believe this is indicative of improved organ oxygenation through enhanced vascular permeability. These results must be weighed against the great variability of the measurements made at given times during the experiment. The increase in lactates throughout the hypothermic preservation phase in the HMP group could be due to a “washing” benefic effect on the organ during HMP in the preservation solution. An alternative hypothesis posits that the temperature of the organ during HMP surpasses that observed during the SCS (due to heating of the medium as it passes through the circuit), which could be deleterious to the organ. In the end, the last hypothesis could envisage a deleterious effect on the organ through hyperpressure of the machine in the vessels. The most plausible hypothesis would appear to be the first, given the opposite tendency for lactates in normothermia and similar descriptions in the literature of other organs when HMP is used.^34^ Monitoring of biochemical parameters during the normothermia phase showed an increase in their concentrations over time in both groups. This increase was also observed in Padma’s study, using a closed normothermic reperfusion system from a preclinical sheep model. The increase in lactate levels during reperfusion is a consequence of the release of lactate produced during preservation.^22^ Regarding the evaluation of ATP concentration, this study shows no significant difference between the 2 groups. Only one study found in the literature on uterine preservation evaluated tissue ATP concentrations in the myometrium of the premenopausal human uterus in SCS according to different preservation fluids. ATP levels showed great variability between control samples, ranging from 0.35 to 11.29 nmol/mg protein, of the same order of magnitude as the results obtained in our study. This above-mentioned study revealed an increase in ATP levels in a suitable preservation fluid compared with Ringer acetate as early as 6 and 12 h of storage and no difference in tissue ATP concentration over time between the preserved liquids.^35^ Similarly, our study was unable to show a difference in tissue ATP concentration between the 2 groups, using only preservation fluids.

This feasibility study also has several limitations. Larger study groups should be set up to refine the results of the secondary endpoints. Variability in the results of secondary endpoints may also be linked to variability in the stage of the hormonal cycle at the time of surgery. Indeed, we know the influence of the hormonal cycle on endometrial thickness. The latter may influence the oxygen requirements of the graft, inflammation, endometrial tissue lesions observed, and the expressivity of endometrial cells. It would be advisable to synchronize the hormonal cycle by administering progesterone over several days, a procedure that was too laborious and time consuming for our study.

Further studies should be required to evaluate these preliminary results in preclinical models of retransplantation or posttransplant birth. Eventually, the study did not include an evaluation of different oxygenation rates for uterine grafts in HMP. Indeed, hypothermic oxygenated perfusion is the subject of various research studies in renal and pancreatic transplantation, comparing different saturations of preserving fluids.^31,36,37^ More studies could consider the evaluation of different oxygenation contributions during the hypothermic preservation phase or the contributions of substrates to enrich the preservation medium, with or without HMP.

## Supplementary Material


