# Supplementary material for: Uterine Transplant Optimization From a Preclinical Donor Model With Controlled Cardiocirculatory Arrest
Source: Transplant Direct. 2024 Dec 10;11(1):e1735. doi: 10.1097/TXD.0000000000001735 (PMC11634325; doi:10.1097/TXD.0000000000001735)

Figure S1 : Differential expression of HMOX-1, VEGF, ICAM-1, VCAM-1 and THBD genes, depending on the study group, in the uterine body during the hypothermic preservation phase

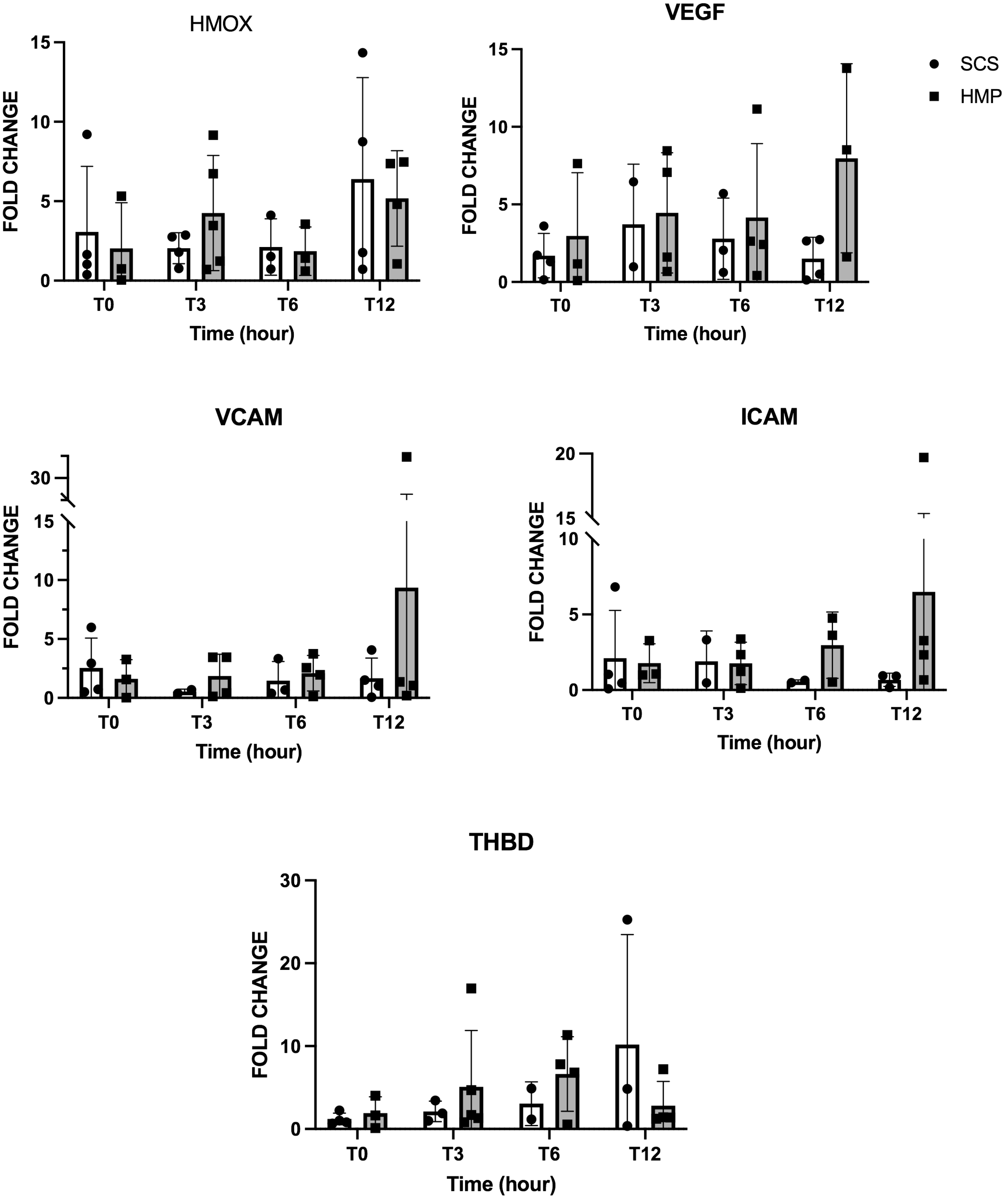

Figure S2 : Differential expression of HMOX-1, VEGF, ICAM-1, VCAM-1 and THBD genes, depending on the study group, in the uterine horn during the hypothermic preservation phase

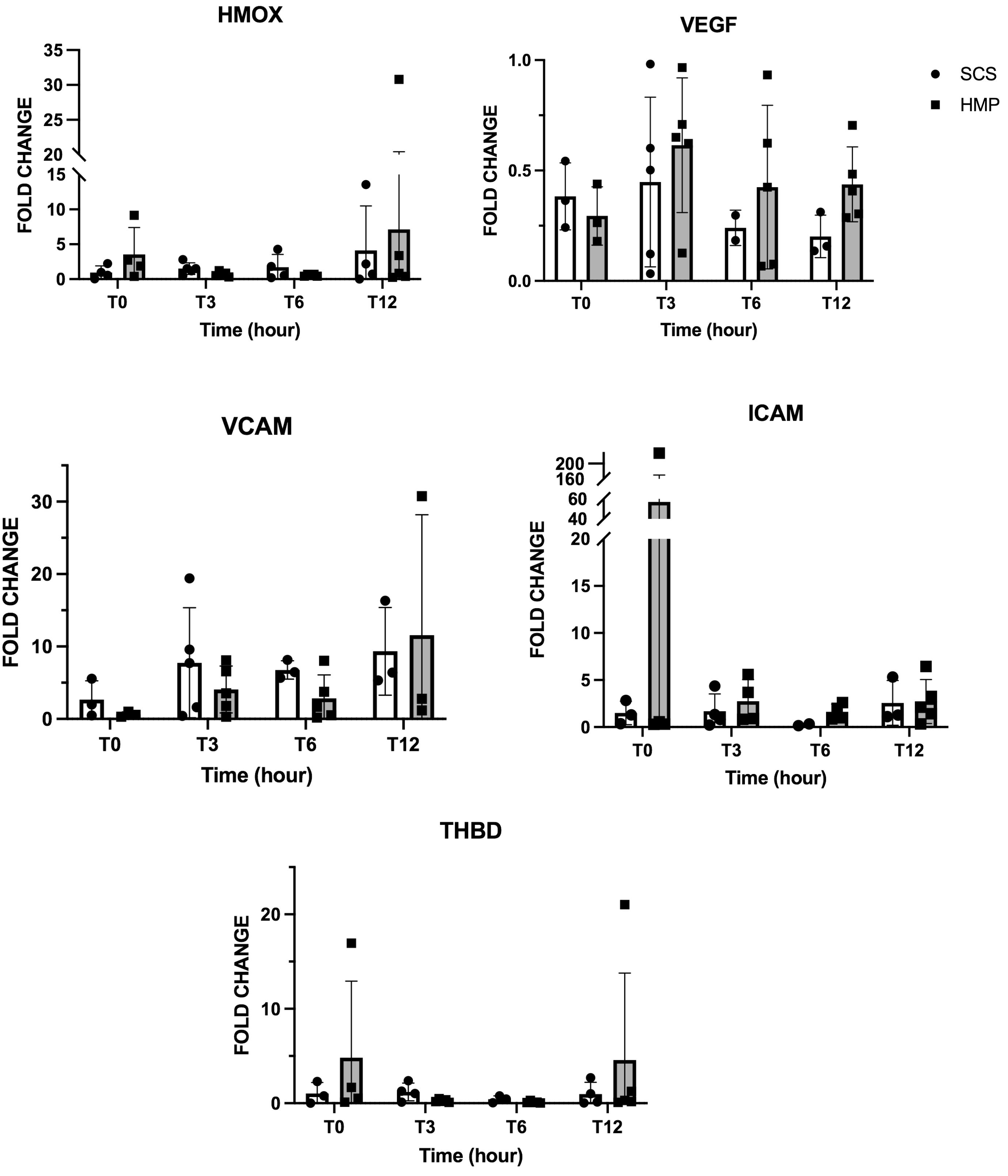

Figure S3 : Differential expression of HMOX-1, VEGF, ICAM-1, VCAM-1 and THBD genes, dependi ng on the study group, in the uterine body during the normothermic reperfusion

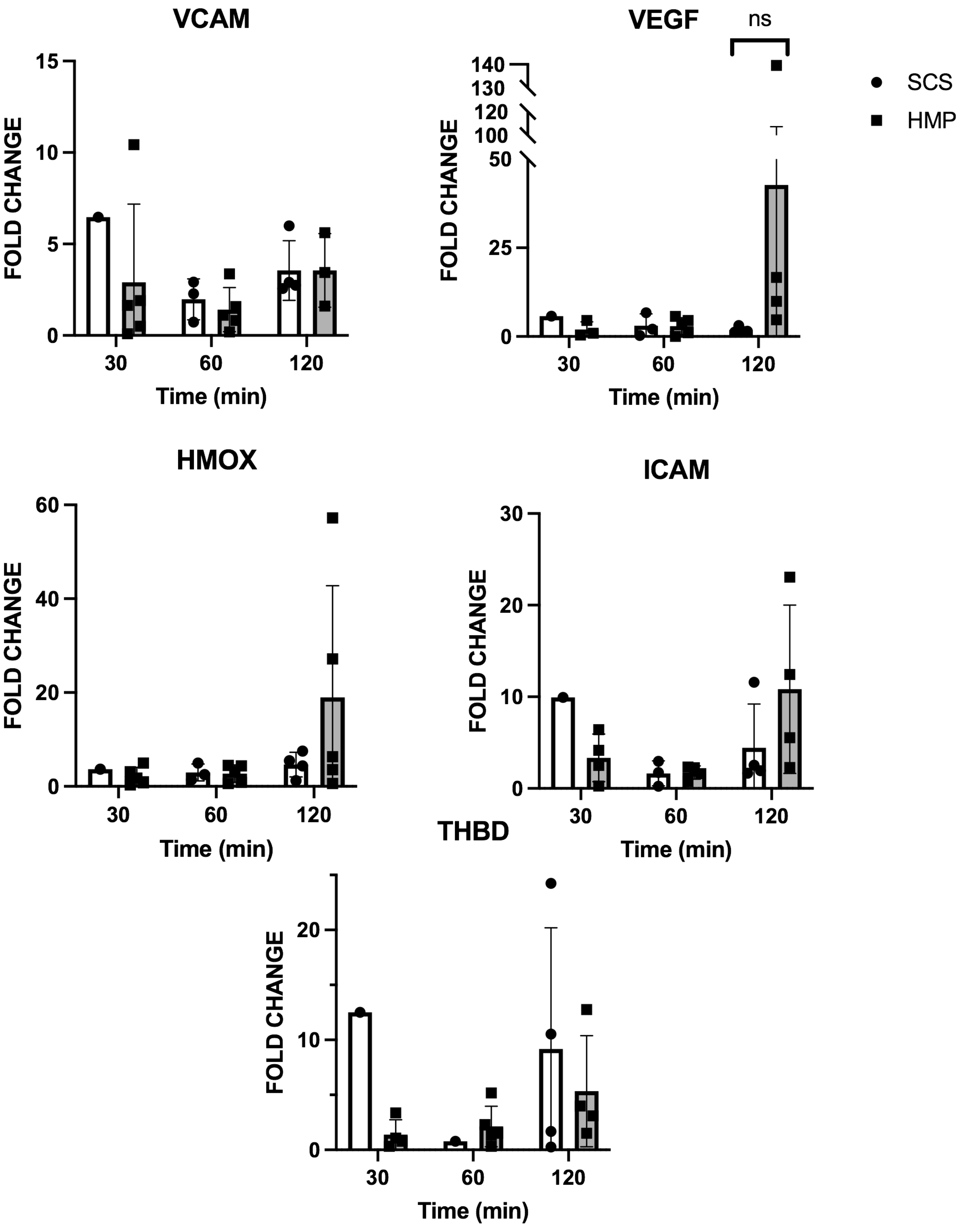

Figure S4 : Differential expression of HMOX-1, VEGF, ICAM-1, VCAM-1 and THBD genes, depending on the study group, in the uterine horn during the normothermic reperfusion

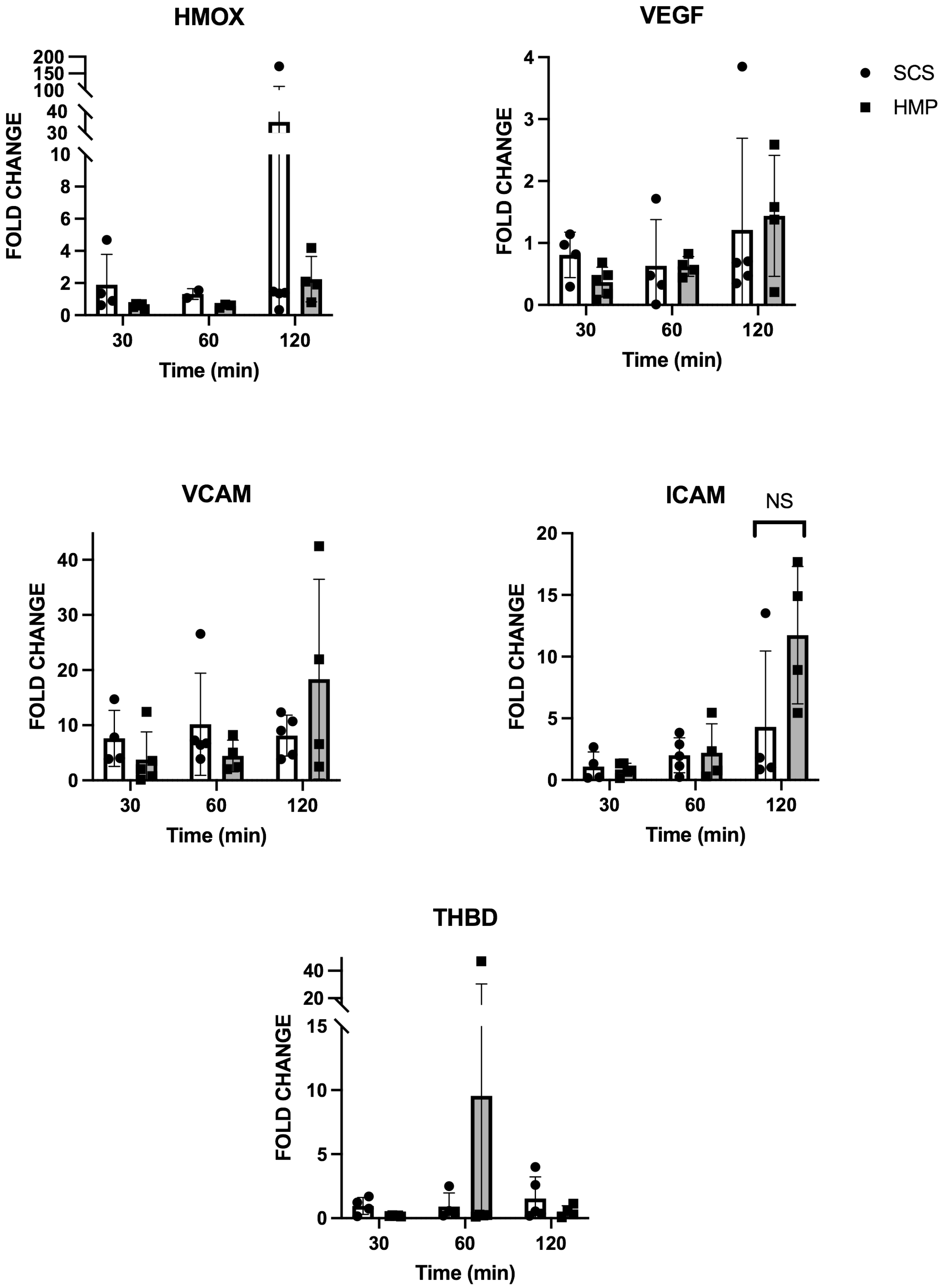

Supplement: Supplementary file 1 [file txd-11-e1735-s001.pdf]
